# Replacement of Corn with Different Levels of Wheat Impacted the Growth Performance, Intestinal Development, and Cecal Microbiota of Broilers

**DOI:** 10.3390/ani14111536

**Published:** 2024-05-23

**Authors:** Luxin Liu, Zilin Wang, Bin Wei, Leilei Wang, Qianqian Zhang, Xuemeng Si, Yanqun Huang, Huaiyong Zhang, Wen Chen

**Affiliations:** 1 Key Laboratory of Animal Biochemistry and Nutrition, College of Animal Science and Technology, Ministry of Agriculture, Henan Agricultural University, Zhengzhou 450002, China; 13592192035@163.com (L.L.); 18437602146@163.com (Z.W.); weibin3046@163.com (B.W.); wll911152@163.com (L.W.); 19337620735@163.com (Q.Z.); sxmswun@126.com (X.S.); hyanqun@aliyun.com (Y.H.); 2 Laboratory for Animal Nutrition and Animal Product Quality, Department of Animal Sciences and Aquatic Ecology, Ghent University, 9000 Ghent, Belgium

**Keywords:** wheat replacing corn, intestinal morphology, microbiota, growth performance, broilers

## Abstract

**Simple Summary:**

In this study, the effects of different levels of wheat inclusion on growth performance and intestinal homeostasis in broilers were evaluated. We first analyzed the effects of dietary wheat ratio on liver function, serum glycolipid profile, and bone turnover, and showed that the dose of wheat in the diets of broilers had no deleterious effects on animal health. We also found that replacing corn with 55% wheat in the diet of broilers increased body weight and feed consumption, with improved intestinal morphology and cecal microbiota composition. These data indicated that the replacement of corn with 55% wheat in the diet promoted the growth performance of 21-day-old broilers via improving intestinal morphology and cecal microbiota.

**Abstract:**

Replacing corn with different levels of wheat in the iso-energy and -protein diet of broilers and the impacts on growth performance and intestinal homeostasis of broilers under the condition of supplying the multienzyme complex were evaluated in this study. A total of 480 10-day-old male broilers were assigned randomly to the low-level wheat group (15% wheat and 35.18% corn), the medium-level wheat group (30% and 22.27%), and the high-level wheat group (55.77% wheat without corn) until 21 d. The different levels of wheat supplementation did not affect hepatic function, serum glycolipid profile, or bone turnover. The replacement of corn with 55% wheat in the diet of broilers increased the body weight at 21 d and feed intake during 10 to 21 d (both *p* < 0.05), with a comparable feed conversion ratio. Compared with the low-wheat group, the dietary addition of medium or high wheat levels notably increased the ratio of villus height to crypt depth in the duodenum (*p* < 0.05) and the ileal villus height (*p* < 0.05). Meanwhile, the supplementation of medium and high wheat in the diet increased the proportion of Bacteroidetes, and a diet with high wheat proportion elevated the content of Firmicutes when compared to the low-level wheat group (both *p* < 0.05). In addition, the diet containing 30–55% wheat enhanced the anti-inflammatory capability in both the ileum and the serum. These findings suggest that the replacement of corn with 55% wheat in the diet improved the growth performance of 21-day-old broilers, which might be linked to the alteration in intestinal morphology and cecal microbiota.

## 1. Introduction

The poultry industry faces constant challenges related to feed costs and the nutritional requirements of broiler chickens. The supply of feed represents most of the total production cost of broilers in the poultry industry, as it contributes to up to 70% of the total production cost [1]; thus, reducing the feed costs is essential to improve the economic benefits of broiler farming. Corn is commonly used in the preparation of feed as an energy source in broiler production worldwide [2], but the high demand and price volatility of corn have led to the exploration of alternative feed sources. In this context, wheat could be considered as a suitable replacement for corn in the diets of broilers [3], because it is widely used as energy feed for livestock in Europe and it possesses a similar nutritional profile to corn [4]. Nevertheless, wheat contains lower starch than corn, but it has higher crude protein and amino acid levels, including lysine, tryptophan, and threonine [5]. In addition, wheat has a relatively higher phosphorous digestibility and total calcium concentration than corn in livestock [6,7], which implies that the supplementation of wheat might possess higher bioavailability of phosphorus and calcium. Of note, the contents of non-starch polysaccharides (NSPs) in wheat are higher than in corn [8], which results in increased viscosity, thereby separating substrates from endogenous enzymes and consequently decreasing nutrient utilization [9]. The higher fiber content in the wheat–soybean diet might encapsulate other nutrients to shield them from digestive enzymes and might therefore reduce their digestibility [10], resulting in the poor digestibility of crude protein and ether extract [11,12], thus compromising growth performance. This data indicates the importance of optimal replacement levels of corn with wheat in the diet in consideration of feed costs and the growth performance of livestock.

Regarding the effects of wheat on the growth performance of domestic animals, the data from the growing–finishing pigs showed that the diet with different levels of wheat did not affect the growth performance of pigs [13]. A study on broilers found that broilers fed wheat-based diets had a lower body weight (BW) and a lower feed conversion ratio (FCR) compared to those fed corn-based diets [14]. However, another previous study showed that it resulted in better growth performance in the broilers fed wheat diets than those consuming corn diets [15]. Moreover, a previous study indicated that the increase in the viscosity of the chyme due to the presence of NSP in wheat reduces the rate of diffusion of substrates and digestive enzymes and inhibits their effective interactions, which is detrimental to the growth of broilers due to a decrease in the digestion and absorption of nutrients [16]. In practice, a previous study has shown that the NSP-degrading enzyme supplementation in broiler feed reduces the digesta viscosity and releases encapsulated nutrients [17]; thus, the application of NSP-degrading enzymes in wheat-based diets is necessary to improve the weight gain of broilers. In addition, because the gastrointestinal tract of broilers is highly sensitive to dietary changes, improper nutrition can lead to digestive disorders and subsequent growth retardation. Hetland and colleagues found that a whole wheat diet was found to promote the enlargement and development of the gizzard in broilers [18]. In comparison to the corn diet, feeding the wheat diet was observed to increase the weight of the cecum and the length of both the duodenum and the ileum in the broilers [19]. Overall, despite the great potential of wheat to replace corn, the impacts of different levels of wheat substitution for corn on the growth performance and intestinal health of broilers remain to be illustrated given the nutritive composition and high NSP content.

Therefore, the objectives of the current study were to evaluate the effects of wheat as a replacement for corn in the iso-energy and -protein diet of broilers on the growth performance, intestinal development, and cecal microbiota of broilers under the condition of supplying the multienzyme complex. The findings from this study are expected to provide valuable insights into the optimal level of wheat substitution for corn in broiler diets, thus leading to cost-effective production while maintaining broiler production and animal welfare.

## 2. Materials and Methods

### 2.1. Ethics Statement

All procedures of the present experiment were approved by the animal care committee of Henan Agricultural University (No. HNND20190306).

### 2.2. Birds, Diets, and Management

In this study, a total of 480 1-day-old AA broilers were purchased from the local commercial hatchery and fed the same diet to 10 d of age. Subsequently, the broilers with similar BWs were randomly allotted to one of three dietary treatment groups. The experimental diets included the low-level wheat group (15% wheat and 35.18% corn), the medium-level wheat group (30% and 22.27%), and the high-level wheat group (55.77% wheat without corn). Each treatment contained 8 replicate cages (20 chickens/cage). The diets were formulated according to the nutritional requirements of AA broilers, in which the complex enzymes, including xylanase, cellulase, β-glucanase, mannase, pectinase, and α-galactosidase, were added to the experimental diets (Table 1). The trial started from the age of d 11 to 21. The temperature was maintained at 34 °C on d 1 and gradually decreased to 22 °C by d 21 of age. The lighting program was 23 L:1 D during the experiment period. Birds had ad libitum access to feed and water. BW was registered by cage at d 10 and d 21. Feed intake (FI) and the number of dead birds were recorded from d 10 and 21 on a replicate basis. The weight gain, feed conversion rate expressed as the ratio of feed consumption to gain (F:G), and mortality were calculated throughout the experimental period.

### 2.3. Sample Collection

At 21 d, one bird was selected according to the average BW of each pen for sampling. Blood was collected from the jugular vein and by separating the serum. Subsequently, these birds were euthanized through anesthetization and exsanguination, and the liver, gizzard, spleen, thymus, and bursa of Fabricius were weighed to calculate the relative weight. The gastrointestinal tract with contents of all broilers was immediately removed, and the duodenum (from the pyloric junction to the most distal point of insertion of the duodenal mesentery), jejunum (from the most distal point of insertion of the duodenal mesentery to Meckel’s diverticulum), and ileum (from Meckel’s diverticulum to the ileocecal junction) were separated. After measuring the intestinal length, the weight was obtained after removing the chyme. In particular, the right and left ceca were dissected, and the weight and length of the ceca were calculated based on their average. Segments of 1 cm length from the middle of the duodenum, ileum, and jejunum, as well as the liver tissue (around 1 cm^3^), were dissected and immersed in 4% phosphate-buffered formaldehyde for histological analysis. The mid-ileum (by removing 1 cm right in the middle for histological analysis) was collected to gather the mucosa. Cecal contents were obtained for 16S rRNA microbiome sequencing.

### 2.4. Intestinal and Liver Morphology Determination

**Figure 1:**
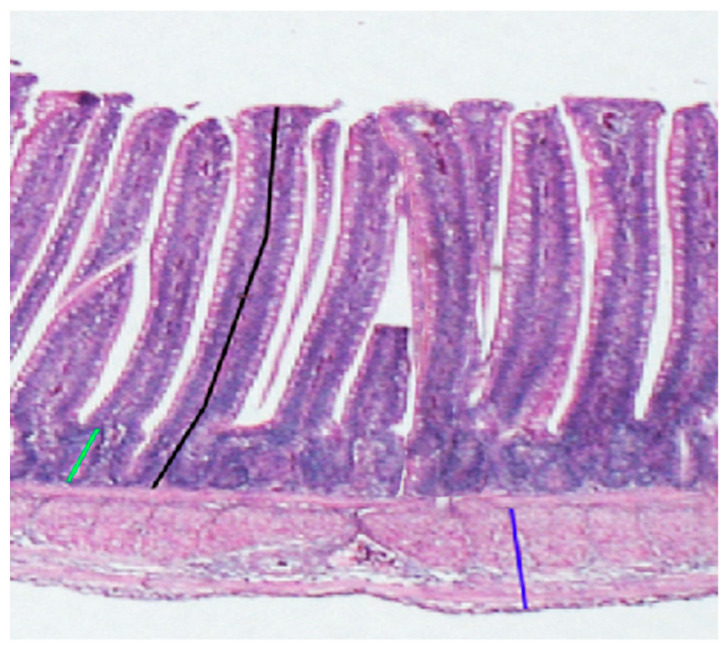
Morphometric measurements of villus height (black bar), crypt depth (green bar), and muscular thickness (dark blue bars) on a jejunum from a broiler chick (hematoxylin and eosin staining, 100×).

The liver, duodenum, jejunum, and ileum segments fixed in formalin solution for one week were dehydrated and embedded in paraffin. The slices were subjected to staining with hematoxylin–eosin (H&E). For intestinal analysis, the villus height, crypt depth, and muscular thickness were measured in at least 10 well-oriented villi, according to Figure 1. The ratio of villus height to crypt depth was then calculated.

### 2.5. Serum Biochemistry

The serum concentrations of various compounds, including calcium (Ca), phosphorus (P), alanine aminotransferase (ALT), aspartate aminotransferase (AST), total protein (TP), albumin (ALB), globulin (GLB), glucose (GLU), triglyceride (TG), cholestenone (CHO), low-density lipoprotein (LDL-c), and high-density lipoprotein (HDL-c), were analyzed using an automatic biochemical analyzer (Beckman Coulter AU5800, California, USA), with corresponding commercial kits. The concentrations of interleukin (IL)-1β, IL-6, IL-10, tumor necrosis factor-alpha (TNF-α), and transforming growth factor beta (TGF-β) were determined using commercial kits following the manufacturer’s instructions. Serum immunoglobulin (Ig) analyses, including IgG, IgA, and IgM, were analyzed using commercial chicken-specific enzyme-linked immunosorbent assay (ELISA) kits. Bone turnover markers in serum, including alkaline phosphatase (ALP), procollagen type I N-terminal propeptide (PINP), and C-terminal cross-linked telopeptide of type I collagen (CTx), were quantified with available kits. All of the kits were purchased from Nanjing JIANCHENG Bioengineering Institute (Nanjing, China). Of note, the serum samples were run in duplicate for confirmation of accuracy.

### 2.6. Ileum Gene Expression

Intestinal barrier and inflammation-related gene expressions were determined using RT-qPCR. Total RNA was extracted from the ileal mucosa. After evaluating the quality and concentration by using the Nanodrop (Thermo Fisher Scientific™, Waltham, MA, USA), the cDNA was synthesized using a cDNA reverse transcription kit (Takara, Dalian, China), and then gene expression analysis was performed using SYBR green qPCR master mix (Takara, Dalian, China). The amplification conditions consisted of an initial denaturation step at 95 °C for 15 s, followed by 40 cycles of amplification at 95 °C for 30 s and 60 °C for 34 s, with a final melting curve analysis. The expression was quantified by normalizing the expression β-actin, which was used as an internal reference gene. Broiler-specific primers are displayed in Table 2.

### 2.7. Cecal Microbiota Analysis through 16S RNA

The total DNA was extracted from the ceca contents and purified using the Zymo Research BIOMICS DNA Microprep Kit. The extracted DNA was detected for quality using 0.8% agarose gel electrophoresis. The V4 region included former primers 515F (5′-GTGYCAGCMGCCGCGGGTAA-3′) and reverse primers 806R (5′-GGGACTACHVGGGTWTCTAAT-3′). The two-ended sequences were spliced using FLASH. Each sample sequence was then isolated from raw reads based on the barcode using QIIME (version 1.9.0), with the barcode sequence truncated. The UPARSE algorithm was used to cluster the operational taxonomic units (OTUs) at a 97% threshold value. The alpha diversity was estimated with the ACE and Simpson indexes, and beta diversity at the genus level was estimated by calculating Bray–Curtis dissimilarity and visualized with principal coordinates analysis (PCoA). The alterations in microbial composition following 16S rRNA amplicon sequencing were determined in R using the MicrobiotaProcess package for community analysis (version 3.17).

### 2.8. Statistical Analyses

Data were expressed as means ± standard deviation and analyzed using the JMP software, version 13 (SAS Institute, Cary, NC, USA). Data were checked for the normal distribution and homogeneity of variance, and the mortality was analyzed using the Kruskal–Wallis test followed by Dunn’s multiple comparisons. The statistical differences of the rest of the biological parameters were detected via a one-way repeated measure analysis of variance (ANOVA) with Tukey’s post hoc comparison. *p* < 0.05 was considered statistically significant.

## 3. Results

### 3.1. Growth Performance

The effects of different levels of wheat supplementation on broiler growth performance are shown in Table 3. When compared to the low and medium wheat groups, the incorporation of high wheat in diets significantly increased the BW at 21 d, as well as weight gain and FI during 1 to 21 d (both *p* < 0.05), and thus failed to change the F:G relative to the low and medium wheat groups. There was no obvious difference in terms of survival proportions among experimental groups (Table 3).

### 3.2. Alterations in the Gastrointestinal Tract

The effect of dietary treatments on digestive organ development is presented in Table 4. The partial replacement of corn with wheat in the diet failed to alter the absolute weight of the glandular stomach, gizzard, small intestine, and ceca. However, the birds fed the high-level wheat diet displayed a decreased relative weight of the jejunum when compared to those receiving the low-level wheat diet (*p* < 0.05). Meanwhile, the length of the intestine was examined, and it was found that the dietary wheat supplementation did not apparently change the absolute or relative length of the duodenum, jejunum, ileum, or ceca of 21-day-old broilers (*p* > 0.05; Table 4).

**Figure 2:**
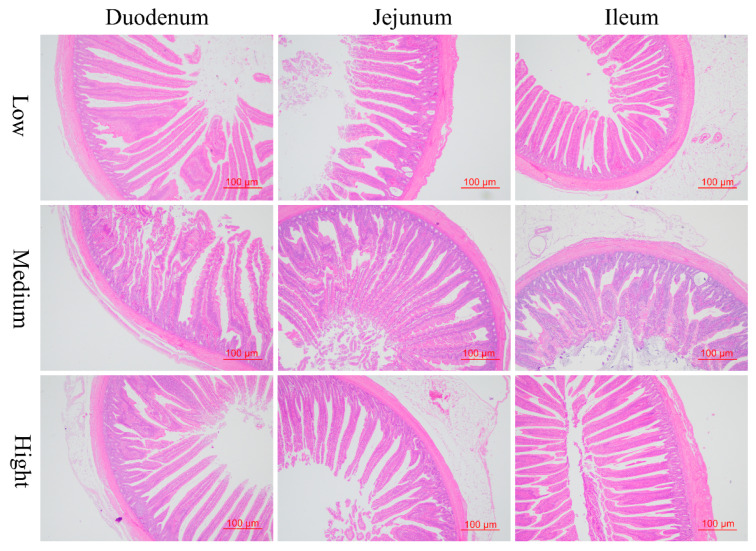
The morphometric alteration of the small intestine response to the replacement of corn with different levels of wheat in the 21-day-old broilers based on the hematoxylin and eosin (H&E) staining (100×).

As illustrated in Figure 2 and Table 5, although there was no significant difference in terms of the villus height, crypt depth, and muscle thickness of the duodenum, the dietary addition of medium and high wheat levels notably increased the ratio of villus height to crypt depth in duodenum compared with the low wheat group (*p* = 0.035), which implies the improvement of duodenal absorption and integrity due to the supplementation of medium and high wheat levels in the basal diets. However, the high level of substitution of wheat for corn significantly increased (*p* < 0.05) the crypt depth of the jejunum with decreased villus height (*p* > 0.05) relative to the medium-level wheat group, resulting in the notably lower ratio of villus height to crypt depth (*p* < 0.05). Meanwhile, the broilers that consumed the medium content of wheat had increased muscular thickness compared to those fed the low-level wheat diet (*p* < 0.05). Considering the morphotype of ileum, with the increasing wheat levels in diets, the villus height was increased when compared to the low wheat diets (*p* < 0.05; Table 5). The ileal crypt depth was increased by the medium-level wheat diet in comparison with the high- and low-level wheat groups (*p* < 0.05). Accordingly, a diet with high wheat content contributes to an increased villus height to crypt depth ratio in the ileum compared to the medium-level wheat diet (Table 5).

**Figure 3:**
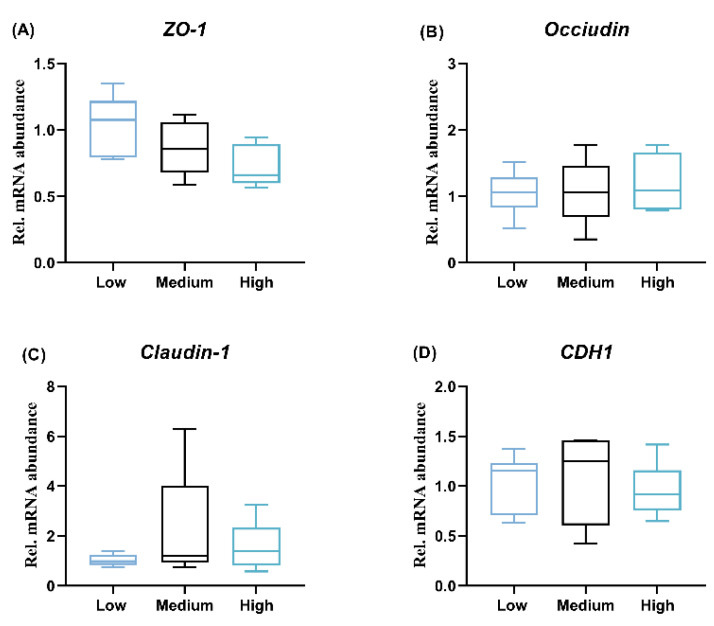
Intestinal barrier function in response to dietary wheat supplementation. (**A**) The mRNA level of Zonula occludens-1 (ZO-1), (**B**) Occiduin, (**C**) claudin, and (**D**) cadherin 1 (CDH1) in ileum mucosa. In the box–whiskers plots, boxes are bounded by the 25th and 75th percentiles, with the median shown by the line bisecting the box. Whiskers extend to the full range of the data. The statistical differences of the rest of the biological parameters were detected via a one-way repeated measure analysis of variance with Tukey’s post hoc comparison (*n* = 8). *p* < 0.05 was considered statistically significant.

In addition, the outcomes of RT-qPCR showed that no significant difference was observed regarding the transcription of zonula occludens-1 (*ZO-1*), cadherin 1 (*CDH1*), *claudin-1*, and *occludin* among the three groups in the ileum (Figure 3A–D).

### 3.3. Cecal Microbiota Composition

**Figure 4:**
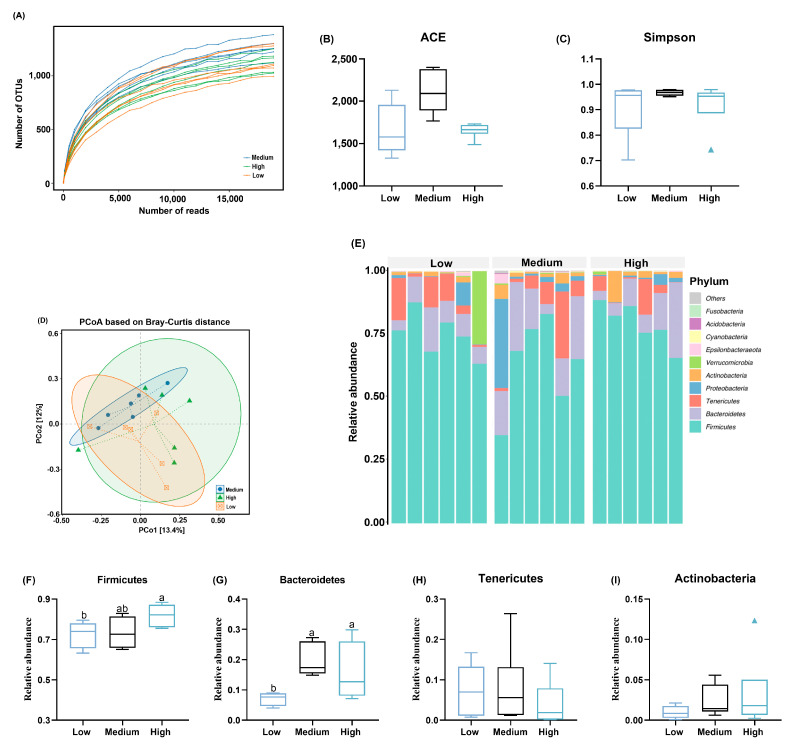
Effects of the partial substitution of corn by wheat on the cecal microbiota of broilers. (**A**) The dilution curve shows the trend of species richness with sequencing depth. (**B**,**C**) Alpha diversity was evaluated via ACE and Simpson indices. (**D**) Principal coordinate analysis plot (PCoA) of cecal microbiome diversity based on Bray–Curtis distance. (**E**) Composition of microbiota at the phylum level in cecal contents of broilers. (**F**–**I**) The abundance of Firmicutes, Bacteroidetes, Tenericutes, and Actinobacteria. In the box–whiskers plots, boxes are bounded by the 25th and 75th percentiles, with the median shown by the line bisecting the box. Whiskers extend to the full range of the data. Outliers are represented by dots. Different letters in the same column represent the significant difference at *p* < 0.05 (*n* = 6).

Figure 4A shows that the flora richness of each sample increased with the increase in the sequencing depth, and the end of the curve tended to be flat, indicating that the amount of sequencing data was reasonable. However, the supplementation of different levels of wheat did not have a significant impact on alpha-diversity measures, such as ACE and Simpson indices (*p* > 0.05; Figure 4B,C). The beta-diversity analysis based on PCoA revealed a clear separation between the three groups (Figure 4D), where PCoA1 and PCoA2 accounted for 13.4% and 12.0% of the variation in microbial diversity, respectively, indicating that the dietary supplementation of wheat altered the composition of ceca microbiota. The abundances of the top 10 phyla are presented in Figure 4E, with Firmicutes and Bacteroidetes being the dominant phyla in the cercal microbiota. The high-level wheat group had a higher (*p* < 0.05) relative abundance of Firmicutes relative to the low-level wheat group (Figure 4F), and supplementation with medium and high levels of wheat in the diet increased the proportion of Bacteroidetes compared to the low-level wheat group (*p* < 0.05; Figure 4G). With the increasing wheat proportion, the content of Tenericutes and Acidobacteria gradually reduced and increased in the ceca microbiota of broilers (both *p* > 0.05; Figure 4H,I), respectively.

### 3.4. Liver Health

**Figure 5:**
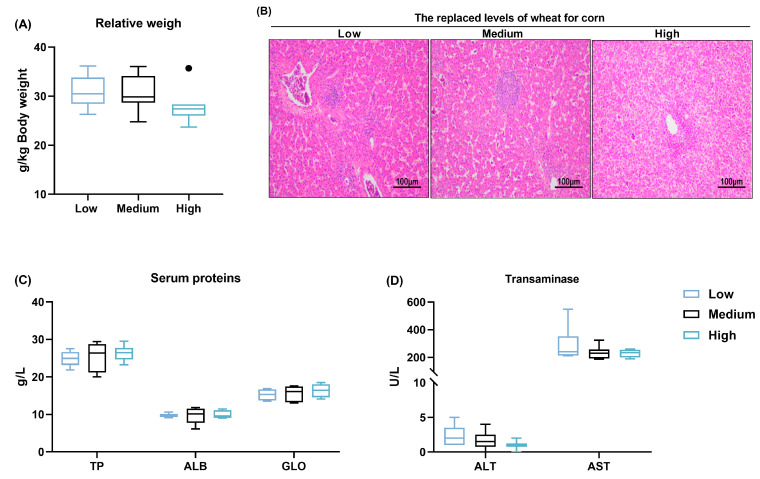
Influence of partial replacement of corn with wheat on liver function of broilers. (**A**) The relative weight of the liver. (**B**) Micrograph of liver microstructure (H&E staining, 100×). (**C**) Serum content of total protein (TP), albumin (ALB), and globulin (GLB). (**D**) Serum level of transaminase, including alanine aminotransferase (ALT) and aspartate aminotransferase (AST). Values are means and standard deviation represented by vertical bars in a scatter plot. In the box–whiskers plots, boxes are bounded by the 25th and 75th percentiles, with the median shown by the line bisecting the box. Whiskers extend to the full range of the data. Outliers are represented by dots. *p* < 0.05 was considered statistically significant (*n* = 8).

No significant differences (*p* > 0.05) were observed in the relative weight of the liver in response to the different wheat levels in the diet (Figure 5A). Histological analysis showed normal hepatocytes without necrosis. The cells were arranged in order, and the nuclei were large and round with even cytoplasm across all treatment groups. With the increase in dietary wheat content, the hepatocyte boundary increased, and a great deal of lipid droplets were observed in the cell plasma (Figure 5B).

Reflecting on the serum biochemical indexes, the high replacement of wheat for corn did not alter the serum TP, ALB, or GLO levels when compared with the medium wheat diet group (Figure 5C). There were no significant differences in terms of the activities of AST or ALT among the three groups (Figure 5D). These results indicate that the replacement of corn with different levels of wheat for 11 d does not impair the hepatocellular function.

### 3.5. Inflammatory Status

**Figure 6:**
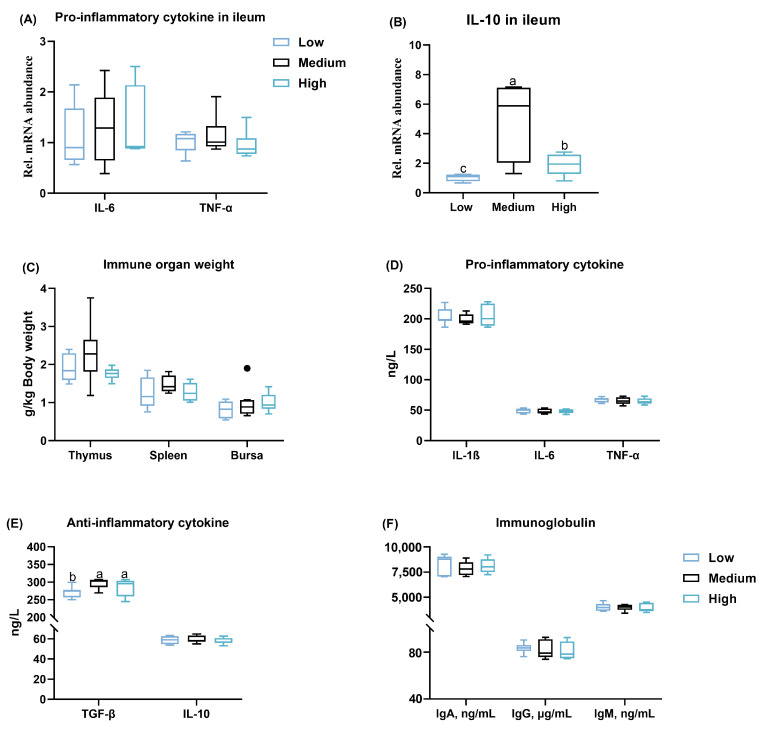
Influence of the partial replacement of corn by wheat on the immunity of 21-day-old broilers. The relative mRNA abundance of pro-inflammatory cytokines (**A**) interleukin (IL)-6 and tumor necrosis factor-alpha (TNF-α), as well as (**B**) anti-inflammatory cytokine IL-10 in the ileum. (**C**) Relative weight of thymus, spleen, and bursa. (**D**) Serum concentration of pro-inflammatory cytokines, including IL-1β, IL-6, and TNF-α. (**E**) The content of anti-inflammatory cytokines IL-10 and transforming growth factor beta (TGF-β) in serum. (**F**) Serum immunoglobulin (Ig) levels. Values are means and standard deviation represented by vertical bars in a scatter plot. In the box–whiskers plots, boxes are bounded by the 25th and 75th percentiles, with the median shown by the line bisecting the box. Whiskers extend to the full range of the data. Outliers are represented by dots. Different letters in the same column represent the significant difference at *p* < 0.05 (*n* = 8).

In ileal mucosa, the mRNA levels of pro-inflammatory cytokines, including IL-6 and TNF-α, were not affected by different ratios of wheat in the diet (Figure 6A), whereas medium and high wheat supplementation significantly upregulated the relative mRNA expression of anti-inflammatory factor IL-10 when compared to the low-level wheat group (Figure 6B). In addition, no apparent differences were found regarding the relative weight of the thymus, spleen, and bursa among the three groups (Figure 6C). Moreover, the outcomes of serum inflammatory factor revealed that the dietary wheat inclusion did not change the anti-inflammatory cytokine IL-10, the pro-inflammatory factors IL-1β, IL-6, and TNF-α, or the immunoglobin concentration in the present study (Figure 6D–F), while medium and high levels of wheat increased (*p* < 0.01) the content of serum TGF-β when compared to the low-level wheat diet (Figure 6E).

### 3.6. Serum Glycolipid Profile and Bone Turnover

As presented in Table 6, when compared with the low-level wheat group, the concentrations of GLU, CHO, and TG were comparable among the three groups. There was also no significant effect on the serum levels of LDL-c and HDL-c among the experimental groups (*p* > 0.05). Dietary wheat treatments did not change the content of serum Ca and P in 21-day-old broilers (*p* > 0.05). The outcomes of serum bone formation markers revealed that the concentrations of P1NP and ALP were similar among low, medium, and high groups (*p* > 0.05). Meanwhile, the serum CTx level, representing bone resorption, was not changed by dietary wheat treatments (*p* > 0.05).

## 4. Discussion

It is well-established that corn, serving as the energy resource, is the primary ingredient in poultry diets, which have recently required substitutes due to shortages and rising prices. Multiple studies have provided stronger evidence that wheat might be an excellent option to replace corn in poultry diets [20,21,22]. One of the factors for wheat substitution for corn is the existence of high NSPs, an anti-nutritive factor, in wheat compared to corn, which might lead to adverse effects on the growth performance, intestinal homeostasis, and health status of birds. This highlights that the dose of wheat used in broiler diets is very important. Considering that the goal of wheat utilization is to optimize animal health and food security rather than simple production in the broilers industry, the substitution level of wheat for liver function, glycolipid metabolism, inflammatory status, and bone turnover has been evaluated in the current study. By evaluating the healthy status and function of hepatocytes, the present data suggest that the replacement of corn with less than 55% wheat did not impair liver function, as evidenced by indifferent AST and ALT activity, both biomarkers of early liver injury, and similar serum levels of TP, ALB, and GLO in this study. There is even research showing that wheat malt extracts (100 or 200 mg/kg) reduce the degree of hepatological histological injury and serum ALT and AST in a dose-dependent manner [23]. Therefore, in this experiment, the dose of wheat in the diets of broilers had no deleterious effects on liver health.

The appropriate level of substitution of wheat for corn also manifested in a stable glycolipid profile in serum and bone turnover. Previous studies have found that the supplementation of wheat fermentation products caused the reduction of serum TG and LDL-c and resulted in increased HDL-c [24]. Nevertheless, other outcomes suggested that serum TG, TC, and LDL-c levels were increased significantly after feeding refined wheat flour for 16 weeks, which was accompanied by decreased HDL-c levels in model rats [25]. In the present study, the increase in wheat substitution levels did not alter the contents of GLU, CHO, TG, LDL-c, or HDL-c in serum, indicating that replaced levels of wheat in broiler feed do not affect glycolipid metabolism. These discrepancies among studies might derive from the form of wheat production, the feed materials selected, and the different fermentation capacities of trial objects. Of note, a link between lipid metabolism and bone metabolism is well-known. Guo et al. showed that leg disorders are associated with compromised growth process, bone quality, bone structure, and lipid metabolism [26]. The probable mechanism might be partly due to the common origin of adipocytes and osteoblasts, both originating from mesenchymal stem cells (MSCs) [27]. MSCs present in bone marrow possess the ability to differentiate into osteoblasts and adipocytes, which play a crucial role in bone formation and the adipose tissue component within the marrow, respectively. An inverse relationship between bone marrow adiposity and bone mass has been noticed in bone diseases, such as osteoporosis [28]. In this study, the indifferent serum glycolipid profile might explain the comparable bone turnover, showed by the similar concentrations of P1NP and ALP (both indicating bone formation) and CTx (a biomarker of bone resorption) in serum.

The fact that the higher contents of NSP and fiber in wheat could separate substrates from endogenous enzymes and, consequently, decrease nutrient utilization [9,10], BW, or weight gain response to the wheat-based diets is inconclusive. The published literature points out that the broilers receiving wheat diets exhibited improved weight gain compared to those receiving corn-based diets [15,29], which was further supported by the current study. No apparent effects on the BW were noticed in broilers fed an inclusion of 40% wheat in a corn-based diet [14], and growing–finishing pigs consumed a corn–soybean meal-based diet with 10–60% wheat [13]. The higher wheat levels in the diet were also found to reduce BW and weight gain in broilers when compared to those offered the corn-based diets [30]. This might be explained by the higher levels of NSPs in these diets, which increase the chyme viscosity, thus reducing feed consumption and growth performance [31]. It was reported that the wheat-based diet increased by approximately three times a less viscous ileal digesta in broilers when compared to the corn-based diet [32]. Therefore, the delayed transit time and gut motility due to higher viscosity hindering feed consumption is warranted in higher NSP-containing diets [9]. Outcomes from broilers showed that the supplementation of a wheat-based diet impaired the FI and reduced the FCR content from 25 to 42 d in broilers [14]. However, in this study, the feed consumption of birds of high-wheat diets was higher than those fed other diets, which was accompanied by a similar feed conversion rate. The most probable explanation is the supplementation of enzymes. It was found that incorporating NSP-degrading enzymes, particularly xylanase, into wheat-based diets could damage fiber structures, resulting in a decrease in intestinal viscosity and subsequently increasing nutrient digestibility of birds and promoting foraging [33]. In practice, the NSP-degrading enzyme supplementation in broiler feed reduces the digesta viscosity and releases encapsulated nutrients [17], which highlights the necessity of the application of NSP-degrading enzymes in wheat-based diets for improving the growth performance in livestock and poultry.

Gastrointestinal development is directly related to the absorptive capacity and barrier function of the animals. Feeding a whole wheat diet was found to promote the enlargement and development of the gizzard in broilers [18]. Moreover, a wheat diet was also observed to increase the weight of the ceca and the length of both the duodenum and the ileum in the broilers compared to the corn diet [19]. In the current study, no difference was observed among the treatment groups in the absolute weights of the stomach and the intestine, while the relative weight of the jejunum was decreased in the high-level group of broilers compared to the low wheat group, which is possibly related to the higher concentration of soluble NSP in viscous wheat [34]. Presumably, it seems that an NSP-rich diet (such as a wheat and barley diet) displays a negative effect on gut morphology and integrity because of the higher digesta viscosity [35]. When compared to the corn-based diets, a wheat-based diet was found to induce a decreased villus width in broilers [14], and the feeding of wheat-based diets was shown to reduce the villus height and increase the crypt depth in the jejunum of laying hens [5]. Homoplastically, the increased crypt depth of jejunum compared to the villus height was also noticed in this study. In the literature, a study on growing pigs suggested that the addition of a wheat diet notably increased the villus height and its ratio to crypt depth ratio in the duodenum relative to the corn diet [36]. According to the outcomes of intestinal morphology in this study, the medium- and high-level wheat inclusion in the diet increased the villus height and decreased the crypt depth, thereby increasing their ratio of duodenum and ileum. These beneficial effects of the wheat diet on intestinal morphology in this study probably derive from the supplementation of the complex enzyme because the incorporation of an NSP-degrading enzyme in wheat-based diets remarkably augmented the villus height and reduced the crypt depth of the duodenum in broilers [37]. The improved intestinal morphology might be a contributor to the weight gain of broilers due to the increased absorptive surface area for nutrients. However, this study cannot explain the different responses of intestinal morphological changes to dietary wheat supplementation under the condition of supplying the multienzyme complex, and further research is required to define the relationship between wheat supplements and the intestinal development of broilers.

There is a strong link between intestinal epithelial barrier integrity and systemic inflammatory status. It is well-known that tight junctions are important physical barriers, which are composed of ZO-1, occludin, claudin, etc. In the present study, we did not observe significant differences in the expression of ZO-1, occiudin, claudin-1, or CDH1 in the ileal mucosa, indicating that dietary wheat inclusion failed to notably change the internal integrity of broilers. Surprisingly, medium- and high-level wheat supplementation upregulated the expression of anti-inflammatory cytokine IL-10 in the ileum, although it did not impact the transcription of pro-inflammatory cytokines, including IL-6 and TNF-α. This suggests that dietary 30–55% wheat addition exerted a positive anti-inflammatory role in broilers, and these roles also extend to systemic inflammation, as evidenced by the increased levels of TGF-β in the serum. In agreement with the previous data in broilers [14], the dietary treatments did not alter the relative weight of immune organs, including the thymus, bursa, and spleen, in this study.

To uncover the mechanism underlying the substitution of corn with wheat on the growth performance and intestinal development of broilers, cecal microbiota was detected using 16S RNA, because the higher crude fiber contained in wheat could be directly fermented by microbiota as the energy material [38]. A study on broilers found that the supplementation of wheat-based diets increased the account of *Escherichia coli*, a well-known harmful bacterium, in ileal chyme [14] and decreased the proportion of *Ruminococcin* in the ceca microbiota profile [32] compared to corn-based diets. The decreased account of Firmicutes was also observed in the cecal chyme of pigs fed the wheat diet relative to those who consumed the corn-based diet [36]. These data imply that a diet containing high levels of wheat negatively affects the composition of cecal microbiota. Nevertheless, the outcomes from growing pigs showed that the feeding of wheat-based diets decreased the levels of *Escherichia-Shigella* and increased the proportion of beneficial bacteria, such as *Bifidobacterium* and *Lactobacillus,* at the genus level [36]. In addition, another study on broilers also found that the supplementation of high levels of wheat to the diet significantly reduced the number of harmful bacteria, such as *Clostridium perfringens,* in the ceca [39]. The discrepancy among these studies may imply that the alterations of microbial composition rely on the dietary components, trial objects, environment, etc., and especially dietary fiber levels. It was reported that the gut microbiota diversity was greatly accelerated with the dietary fiber level in broilers [40]. In this study, the high wheat substitution increased the abundance of Firmicutes and Bacteroidetes, both predominant phyla in the cecum of broilers, compared to the low wheat groups. Firmicutes bacteria, including *Ruminococcin*, *Bifidobacterium*, and *Lactobacillus,* produce high amounts of butyrate and propionate and play crucial roles in intestinal development [41]. For instance, butyric acid appears to exert key roles in improving the absorptive surface of the intestinal epithelium by stimulating the proliferation and/or serving as the energy of enterocytes [42]. The result of a previous study indicated that the butyric acid concentration increased by 71% from 14 to 21 d in broilers fed a wheat-based diet [30]. As a result, the increase in the villus height may be due to the increased proportion of Firmicutes and, consequently, the production of butyric acid, although the results of butyric acid are lacking in this study. Moreover, Bacteroidetes play important roles in the degradation of polysaccharides and oligosaccharides [43], and a decreased abundance of Bacteroidetes has been related to inferior morphology and barrier function dysfunction in the intestine of broilers, thereby resulting in unsatisfied growth performance [44]. Therefore, the elevated abundance of Bacteroidetes together with an increased proportion of Firmicutes in the high wheat substitution might contribute to improved intestinal development and growth performance.

## 5. Conclusions

Taken together, under the condition of supplementing exogenous enzymes, the replacement of corn with wheat in the diets of broilers did not alter the hepatic function, serum glycolipid profile, or bone turnover. Although the supplementation of wheat failed to change the ileal tight junction, a diet containing 30–55% wheat upregulated the anti-inflammatory cytokine *IL-10* expression in the ileum and increased the content of TGF-β in the serum. In addition, the replacement of corn with 55% wheat in the diet of broilers increased the feed consumption and weight gain from 10 to 21 d, which might be linked to the alteration in intestinal morphology and cecal microbiota, especially the elevated proportion of Firmicutes and Bacteroidetes in cecal content. These results will shed light on the optimal level of wheat substitution for corn in broiler diets, keeping in mind both cost-effective productions.

## Figures and Tables

**Table 1:** Ingredients and calculated analysis of nutrients in the basal diet (as-fed).

Items	Starter Diet (1–10 d)	Grower Diet (11 to 21 d)		
		Low	Medium	High
Ingredients, %				
Corn	35.18	35.18	22.27	0.00
Wheat	15.00	15.00	30.00	55.77
Flour	15	15	15	15
Soybean oil	1.0	1.0	1.1	1.3
Soybean meal (46%)	20.2	20.2	18.0	14.2
Peanut meal (50%)	3.0	3.0	3.0	3.0
Corn gluten meal (60%)	3.0	3.0	3.0	3.0
Meat meal	2.2	2.2	2.2	2.2
Sodium chloride	0.2	0.2	0.2	0.2
Limestone	0.98	0.98	0.95	0.90
Montmorillonite	0.2	0.2	0.2	0.2
Dicalcium phosphate	1.60	1.60	1.57	1.55
Choline (60%)	0.1	0.1	0.1	0.1
L-lysine HCl (70%)	0.90	0.90	0.95	1.08
DL-Methionine (99%)	0.22	0.22	0.22	0.23
L-Threonine	0.25	0.25	0.27	0.30
Broiler complex enzyme ^a^	0.30	0.30	0.30	0.30
Sodium bicarbonate	0.1	0.1	0.1	0.1
Sodium butyrate (98%)	0.04	0.04	0.04	0.04
Premix ^b^	0.53	0.53	0.53	0.53
Total	100.0	100.0	100.0	100.0
Nutrient content, %				
AME, Kcal/Kg	2950	2950	2950	2950
Crude protein ^c^	21.32	21.31	21.35	21.44
Dry Matter ^c^	86.82	86.82	87.23	87.94
Crude fiber	2.50	2.50	2.45	2.35
Calcium ^c^	0.88	0.88	0.88	0.88
Total phosphorus ^c^	0.67	0.67	0.68	0.68
Available phosphorus	0.45	0.45	0.45	0.45
Digestible lysine ^c^	1.28	1.28	1.28	1.28
Digestible Methionine ^c^	0.50	0.50	0.48	0.51
Digestible tryptophan ^c^	0.19	0.20	0.20	0.21
Digestible threonine ^c^	0.86	0.85	0.86	0.84

^a^ 100 g complex enzyme included 2% xylanase (100,000 IU/g), 0.75% cellulase (8000 IU/g), 0.8% β-glucanase (50,000 IU/g), 0.3% mannase (50,000 IU/g), 0.5% pectinase (30,000 IU/g), and 0.5% α-galactosidase (2000 IU/g). ^b^ Premix provided per kg of diet vitamin A (retinyl acetate), 9985 IU; vitamin D_3_ (cholecalciferol), 4293 IU; vitamin E (dl-α-tocopherol acetate), 39.3 mg; vitamin K_3_ (menadione), 3.975 mg; vitamin B_1_ (thiamine), 4.293 mg; vitamin B_2_ (riboflavin), 9.67 mg; niacin, 62.96 mg; D-pantothenic acid, 21.75 mg; vitamin B_6_ (pyridoxine-HCl), 4.60 mg; vitamin B_12_ (cyanocobalamine), 0.038 mg; folic acid, 2.16 mg; biotin, 0.2 mg; Fe (FeSO_4_.H_2_O), 41.44 mg; Cu (CuSO_4_.5H_2_O), 10 mg; Zn (ZnO), 80.5 mg; Mn (MnO), 85.56; (Ca(IO_3_)_2_), 0.992 mg; and Se (Na_2_O_3_Se), 0.304 mg. ^c^ These values were the measured value.

**Table 2:** The primers for real-time PCR.

Gene	Gene ID	Primer Sequences (5′→3′)	Product Length, bp
*ZO-1*	NM_001256123.2	F: GAACGTGAAGAGGGGGAAGG	101
		R: TTGCCAGGCTCTCTCCAAAG	
*Occiduin*	NM_205128.1	F: CATGCTCATCGCCTCCATC	256
		R: TAGCGCCAGATCTTACTGCG	
*Claudin*	NM_001013611.2	F: GGTATGGCAACAGAGTGGCT	91
		R: CAGCCAATGAAGAGGGCTGA	
*CDH1*	NM_001039258.3	F: AGCCAAGGGCCTGGATTATG	157
		R: GATAGGGGGCACGAAGACAG	
*TNF-α*	NM_204267.2	F: AGTCAGGAGCGTTGACTTGG	68
		R: GCAACAACCAGCTATGCACC	
*IL-10*	NM_001004414.4	F: AGGAGCAAAGCCATCAAGCA	251
		R: TAGCGGACCGAACGTTAAGC	
*IL-6*	NM_204628.2	F: AGGGCCGTTCGCTATTTGAA	72
		R: CAGAGGATTGTGCCCGAACT	
*β-actin*	NM_205518.1	F: GTCCACCGCAAATGCTTCTAA	78
		R: TGCGCATTTATGGGTTTTGTT	

*ZO-1*, zonula occludens-1; *CDH1*, cadherin 1; *IL*, Interleukin-6; *TNF-α*, tumor necrosis factor alpha.

**Table 3:** Effects of the partial substitution of corn by wheat on the growth performance of broilers.

Item	The Replaced Levels of Wheat for Corn			*p*-Value
	Low	Medium	High	
Body weight, kg/bird				
10 d	0.29 ± 0.01	0.29 ± 0.02	0.29 ± 0.01	0.995
21 d	0.92 ± 0.03 ^b^	0.90 ± 0.04 ^b^	0.96 ± 0.02 ^a^	0.011
Weight gain, kg/bird				
10–21 d	0.63 ± 0.02 ^b^	0.62 ± 0.04 ^b^	0.67 ± 0.01 ^a^	0.003
Feed intake/bird				
10–21 d	0.89 ± 0.06 ^b^	0.91 ± 0.02 ^b^	0.99 ± 0.07 ^a^	0.004
F:G kg/kg				
10–21 d	1.41 ± 0.06	1.48 ± 0.11	1.47 ± 0.11	0.271
Mortality, %				
10–21 d	1.25 ± 2.31	0.63 ± 1.77	0.63 ± 1.77	0.766

All of the results are shown as mean ± standard deviation. Means without common superscripts are significantly different at *p* < 0.05 (*n* = 8). In this study, 10-day-old male broilers were assigned randomly to the low-level wheat group (15% wheat and 35.18% corn), the medium-level wheat group (30% and 22.27%), and the high-level wheat group (55.77% wheat without corn) until 21 d.

**Table 4:** Effects of the partial substitution of corn by wheat on the gastrointestinal weight and length of broilers.

Item	The Replaced Levels of Wheat for Corn			*p*-Value
	Low	Medium	High	
Body weight, kg	0.91 ± 0.04 ^b^	0.92 ± 0.05 ^b^	0.99 ± 0.03 ^a^	0.003
Absolute weight, g				
Glandular stomach	6.14 ± 1.13	6.96 ± 1.65	6.95 ± 0.83	0.339
Gizzard	14.29 ± 2.33	14.83 ± 2.32	15.81 ± 2.18	0.416
Duodenum	9.54 ± 1.28	9.64 ± 1.85	9.41 ± 1.95	0.965
Jejunum	18.90 ± 3.88	17.95 ± 2.91	15.52 ± 3.78	0.171
Ileum	16.46 ± 5.00	14.47 ± 2.97	13.34 ± 4.86	0.372
Ceca	14.75 ± 3.15	11.35 ± 3.78	14.86 ± 2.37	0.314
Relative weight, g/kg body weight				
Glandular stomach	6.72 ± 1.06	7.58 ± 1.83	7.07 ± 0.89	0.441
Gizzard	15.69 ± 2.55	16.11 ± 2.41	16.10 ± 2.53	0.929
Duodenum	10.47 ± 1.37	10.47 ± 1.95	9.54 ± 1.89	0.484
Jejunum	20.83 ± 4.70 ^a^	19.50 ± 3.20 ^ab^	15.69 ± 3.37 ^b^	0.036
Ileum	18.09 ± 5.65	15.80 ± 3.56	13.44 ± 4.47	0.158
Ceca	16.14 ± 3.19	13.01 ± 4.48	15.11 ± 2.52	0.097
Absolute length, cm				
Duodenum	25.53 ± 1.98	25.50 ± 3.66	25.10 ± 1.93	0.937
Jejunum	54.73 ± 4.89	55.63 ± 6.59	55.74 ± 4.12	0.916
Ileum	47.50 ± 2.73	51.38 ± 5.58	49.58 ± 5.34	0.282
Ceca	26.03 ± 4.31	23.06 ± 4.99	27.45 ± 5.83	0.235
Relative length, cm/kg body weight				
Duodenum	28.06 ± 2.58	27.75 ± 4.22	25.48 ± 1.69	0.199
Jejunum	60.20 ± 6.66	60.55 ± 8.06	56.60 ± 3.95	0.414
Ileum	52.16 ± 2.94	55.85 ± 6.28	50.30 ± 4.76	0.089
Ceca	28.50 ± 4.12	25.13 ± 5.54	27.81 ± 5.53	0.393

All of the results are shown as mean ± standard deviation. Means without common superscripts are significantly different at *p* < 0.05 (*n* = 8). In this study, 10-day-old male broilers were assigned randomly to the low-level wheat group (15% wheat and 35.18% corn), the medium-level wheat group (30% and 22.27%), and the high-level wheat group (55.77% wheat without corn) until 21 d.

**Table 5:** Effects of the partial replacement of corn with wheat on the intestinal development of broilers.

Intestinal Segment	The Replaced Levels of Wheat for Corn			*p*-Value
	Low	Medium	High	
Duodenum				
Villus height, μm	1538.84 ± 79.69	1630.21 ± 89.03	1655.39 ± 208.03	0.128
Crypt depth, μm	232.99 ± 30.09	206.03 ± 45.84	194.76 ± 32.20	0.075
Villus height to crypt depth	6.70 ± 0.88 ^b^	8.28 ± 1.90 ^a^	8.78 ± 2.17 ^a^	0.035
Muscular thickness, μm	155.08 ± 19.41	163.21 ± 33.25	146.38 ± 12.43	0.389
Jejunum				
Villus height, μm	1288.43 ± 185.81	1403.04 ± 146.06	1296.74 ± 76.08	0.167
Crypt depth, μm	178.16 ± 38.81 ^ab^	160.49 ± 35.91 ^b^	232.87 ± 72.84 ^a^	0.012
Villus height to crypt depth	7.64 ± 2.37 ^ab^	9.21 ± 2.47 ^a^	6.06 ± 1.82 ^b^	0.015
Muscular thickness, μm	102.01 ± 18.17 ^b^	140.74 ± 23.97 ^a^	124.55 ± 27.70 ^ab^	0.004
Ileum				
Villus height, μm	887.09 ± 92.08 ^b^	990.26 ± 76.27 ^a^	1039.25 ± 96.87 ^a^	0.002
Crypt depth, μm	142.67 ± 39.66 ^b^	217.00 ± 66.97 ^a^	143.81 ± 28.65 ^b^	0.002
Villus height to crypt depth	6.68 ± 2.09 ^ab^	4.88 ± 1.24 ^b^	7.51 ± 1.77 ^a^	0.007
Muscular thickness, μm	140.77 ± 26.58	120.66 ± 19.07	131.75 ± 40.02	0.326

All of the results are shown as mean ± standard deviation. Means without common superscripts are significantly different at *p* < 0.05 (*n* = 8). In this study, 10-day-old male broilers were assigned randomly to the low-level wheat group (15% wheat and 35.18% corn), the medium-level wheat group (30% and 22.27%), and the high-level wheat group (55.77% wheat without corn) until 21 d.

**Table 6:** Effects of the partial substitution of corn by wheat on the serum biochemistry of broilers.

Item	The Replaced Levels of Wheat for Corn			*p*-Value
	Low	Medium	High	
Glycolipid profile				
GLU, mmol/L	13.76 ± 1.99	12.36 ± 1.08	12.83 ± 0.92	0.249
CHO, mmol/L	3.35 ± 0.39	3.28 ± 0.65	3.49 ± 0.69	0.829
TG, mmol/L	0.69 ± 0.30	0.67 ± 0.3	0.57 ± 0.19	0.727
HDL-c, mmol/L	2.23 ± 0.24	2.28 ± 0.41	2.27 ± 0.30	0.970
LDL-c, mmol/L	0.98 ± 0.24	0.87 ± 0.24	0.99 ± 0.33	0.702
Bone turnover				
PINP, μg/L	13.91 ± 1.08	13.95 ± 0.88	13.77 ± 1.07	0.929
CTX, pg/mL	195.6 ± 17.25	189 ± 14.10	195.59 ± 18.71	0.673
ALP, U/L	4990.17 ± 3106.61	4622.67 ± 1768.94	4852.17 ± 1752.32	0.962
Ca, mmol/L	2.47 ± 0.09	2.54 ± 0.19	2.51 ± 0.05	0.573
P, mmol/L	2.18 ± 0.06	2.22 ± 0.19	2.03 ± 0.16	0.097

All of the results are shown as mean ± standard deviation. *p* < 0.05 was considered statistically significant (*n* = 8). In this study, 10-day-old male broilers were assigned randomly to the low-level wheat group (15% wheat and 35.18% corn), the medium-level wheat group (30% and 22.27%), and the high-level wheat group (55.77% wheat without corn) until 21 d. GLU, glucose; CHO, cholestenone; TG, triglyceride; LDL-c, low-density lipoprotein; HDL-c, high-density lipoprotein; PINP, procollagen type I N-terminal propeptide; CTx, C-terminal cross-linked telopeptide of type I collagen; ALP, alkaline phosphatase; Ca, calcium; P, phosphorus.

## Data Availability

Data will be made available on request.
